# Family physicians’ continuing professional development activities: current practices and potential for new options

**Published:** 2016-03-31

**Authors:** Elizabeth Lindsay, Eric Wooltorton, Paul Hendry, Kathryn Williams, George Wells

**Affiliations:** 1 Department of Family Medicine, University of Ottawa; 2 Office of Continuing Medical Education, University of Ottawa; 3 Children’s Hospital of Eastern Ontario Research Institute, University of Ottawa; 4 Department of Epidemiology and Community Medicine, University of Ottawa; 5 University of Ottawa Heart Institute, Cardiovascular Research Methods Centre, Ottawa, ON

## Abstract

**Background:**

As part of needs assessment processes, our Faculty of Medicine (FOM) continuing professional development office investigated the differences between physicians who do and those who do not frequently participate in planned group learning to gain insight into their interest in new forms of continuing professional development (CPD).

**Method:**

We sent a 19 item questionnaire to 485 randomly selected physicians of the 1050 family physicians in Eastern Ontario. The questionnaire examined present participation and satisfaction with CPD activities and perceptions regarding the potential impact of those; and appetite for new opportunities to meet their learning needs.

**Results:**

Of the 151 (31%) physicians responding, 61% reported attending at least one FOM group learning program in the past 18 months (attenders) and 39% had not (non-attenders). Non-attenders indicated less satisfaction (p = 0.04) with present opportunities and requested development in newer approaches such as support for self-learning, on-line opportunities, and simulation.

**Conclusions:**

Although there are high levels of satisfaction with the present CPD system that predominantly offers large group learning options, a substantial number of physicians expressed interest in accessing new options such as personal study and on-line resources.

## Introduction

There are significant challenges for family physicians as they strive to access, evaluate and apply the constant flow of new evidence related to provision of optimal patient care. The scope of primary health care is broad and continues to expand as patients present with multiple health concerns and other psychosocial, personal or social issues requiring understanding and attention.^1^ Health care reform in Canada has resulted in changes to the structures of primary health care practice, such as the increase of networks of practices, inter-professional health care teams and use of technology to support patient management processes.^2^ There are acknowledged gaps between what research evidence suggests for optimal care and the care actually provided by health care professionals and health care systems.^3^–^5^ A variety of factors contribute to these gaps.^6^,^7^ For example, physicians often report that they know what to do clinically, but system related issues such as time constraints or access to community referral resources or diagnostic testing create barriers to applying their clinical knowledge. Physicians’ needs for continuing professional development are also changing^8^ as their roles expand beyond clinical knowledge and skills to include team functioning, administration, and use of electronic records to better manage patient health (including maintaining appropriate disease screening schedules, and delivering evidence based chronic disease management recommendations). Making new knowledge in these many spheres accessible through education has become the focus for providers of continuing professional development (CPD).^9^

Providers of traditional CPD have offered formal learning opportunities through conferences, workshops, and small group sessions to support physicians in their on-going learning. A growing body of scientific literature concludes that traditional group learning programs alone, especially where there is a lack of interaction in the program, play a limited role in supporting needed changes in physicians’ practices.^10^ The College of Family Physicians of Canada (CFPC) encourages its members to choose a wide range of learning opportunities, beyond traditional classroom learning, to maintain and expand knowledge and skills.

In addition, Offices of CPD within the Canadian Faculties of Medicine have a mandate to serve the CPD needs of physicians in their regions; accordingly, these education providers, with encouragement from the College of Family Physicians of Canada (CFPC) will need to broaden the scope of their programming to address emerging content priorities and different approaches to enhance learning.^11^ For example, innovation in planning and implementation of non-clinically oriented programs is warranted^12^ and programs often now include content related to topics such as inter-professional care of patients^13^ and use of follow-up systems to improve patient adherence to treatment plans.^14^,^15^

Although CPD opportunities are expanding, CPD providers have little understanding regarding the readiness of physicians to participate. In this context, we conducted this study to gain an understanding of family physicians’ preferred learning activities, their satisfaction with available CPD, and to identify opportunities for innovation in CPD. The scope of this survey included:

- present CPD activities and their potential impact
- differences between those who attend and are satisfied with present offerings compared to those who attended less and were less satisfied
- appetite for new opportunities to meet their learning needs

## Methods

### Design

This study was conducted using a cross-sectional survey. The questionnaire was designed following the National Physician Survey (NPS)^16^ for comparability with supplementary questions. The questionnaire (available from the authors upon request) of 19 items (English only) contained a combination of response types that depended on the nature of the question, including checklists and 5 point scales. For two areas of inquiry (frequency of participation and perceptions of impact of different CPD activities) we chose wording similar to that of the National Physician Survey (NPS) planned for 2010. These questions had been tested in previous surveys and we anticipated a comparison with the 2010 national survey. To determine future directions, we asked participants to indicate the types of CPD activities that the Office of CPD should expand, as well as to rate the most important attributes of present activities.

### Sampling methods and selection

The CT Lamont Primary Health Care Research Centre (CTLC) at the University of Ottawa maintains a complete database of approximately 1050 general and family physicians practicing in the Champlain Local Health Integrated Network (LHIN). This database provided addresses and phone numbers but not email addresses which eliminated the possibility of an electronic survey. We selected a sample of 500 subjects from this database using simple random sampling without replacement methods. This sample size, adjusting for non-response would ensure that the 95% confidence intervals for the estimated proportions in the study would be between +/− 0.05 or smaller. We were exempted from ethics review by the chair of the Ottawa Hospital Research Ethics Board ethics committee, since this needs assessment process was considered a part of regular program planning/quality improvement for the Office of CPD.

To optimize the response rate, we applied limitations that the survey must take no more than seven minutes to complete, must be easy to read and be no more than four pages in length. A convenience sample of six family physicians completed the pre-test of the questionnaire to address readability and issues related to administration of the instrument, such as length of time needed to complete. This led to a reduction from six to four page questionnaire, thus affecting the number and the depth of areas that we could address.

Survey administration, which began in April 2010, followed Dillman’s recommended methods.^17^ The first mailing sent through regular mail, was followed within 10 days with a postcard reminder. The mailed package identified the University of Ottawa as the sender and included a self-addressed envelope that was individually stamped for ease of return. The covering letter offered responders a $25 discount on a future CPD program at the University of Ottawa. Instead of a second full mailing, beginning one month after the first mailing, research staff called office receptionists to enlist their help in reminding physicians of the survey. At that time, we also offered to send another copy, by fax, of the survey and encouraged them to send responses through fax. These phone calls were conducted over a one month period.

### Analysis

Descriptive statistics for the survey questions are summarized as percentages. Group comparison statistical tests were performed using Fisher’s exact tests due to small sample sizes being compared. A *P*-value < 0.05 was considered statistically significant. SAS software (version 9.2. SAS Institute, Cary N.C.) was used to perform the analysis. Analysis of data was blinded in that identifiers used to keep track of responders and their discount vouchers were removed prior to data analysis and further data sets could not be linked to specific responders.

## Results

### Participants

Fifteen of the surveys were returned due to inaccurate addresses or physicians who had left their practices, leaving us a sample size of 485. We received 151 completed surveys (31%). A recalculation of the estimated proportions for this sample size indicated the 95% confidence intervals would be between +/− 0.08 or smaller. Table 1 describes the survey respondent demographics and practice environments.

### Present CPD activities and perceptions of impact

Participants indicated their frequency of participation in specific types of CPD and rated their perception of the impact on clinical practices of these types of activities. Table 2 provides a distinction between most frequent and least frequent activities as well as respondents’ perceptions of impact. Those who responded NA (not applicable) were removed from the analysis. In general, physicians aged 46 and older seemed to participate more frequently in most CPD activities with the exception of “information seeking using evidence-based resources” but none of the differences were statistically significant

### Level of participation in group learning programs

From a list of eight programs, developed or co-developed by the Office of CPD (OCPD), between November 2008 and end of April 2010, respondents identified those that they had attended. Sixty-one percent of our 151 respondents reported attending at least one OCPD program whereas 28% of our random sample of 485 had attended an OCPD program in the same period of time. This over-representation of participation among respondents was anticipated. We expected that attendees would have more interest in this survey than those who did not participate. There were no demographic differences, such as age or gender, between those who had participated in the past 18 months and those who had not.

### Level of satisfaction with existing CPD opportunities

When asked how satisfied they were with CPD activities (they could include any type of activity) that were currently available to them, respondents answered that they were satisfied (62.6% n=77) or very satisfied (10.6% n=13) with current CPD opportunities. It is important to note that 28 (18.5%) cases did not respond to this question. Those who attended at least one CPD program in the roughly eighteen months prior to the survey, were more likely to be satisfied with current CPD opportunities (p=0.04). There were no significant differences with regards to satisfaction with CPD offerings based on the age of respondents.

### Appetite for new CPD opportunities

**Figure 1:**
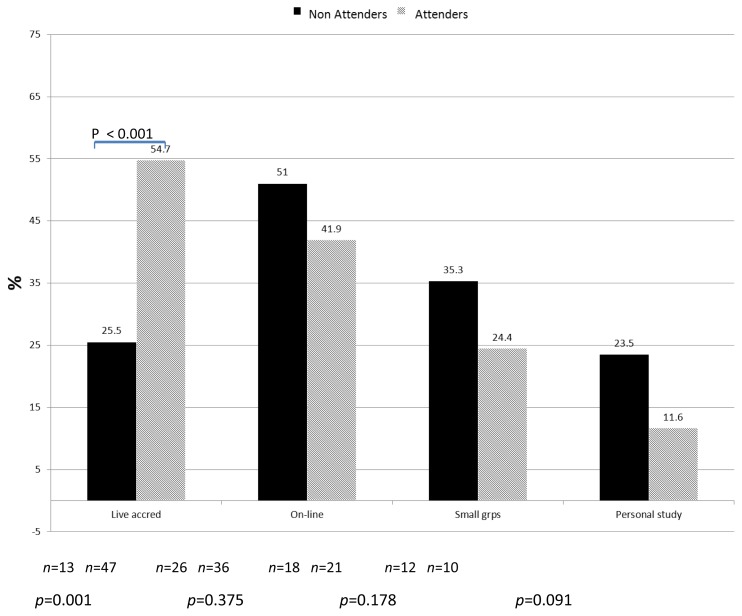
Percentage of attenders (n=86) and non-attenders (n=51) requesting expansion for specific types of CPD. Non-respondents=14

Table 3 describes respondent interest in proposed new CPD offerings. Figure 1 describes differences between attenders and non-attenders regarding recommendations for expansion of CPD activities. Non-attenders indicated statistically significantly (p = 0.001) lower requests for live accredited large sessions or brief didactic presentations. Less satisfied respondents also expressed less interest in more group learning opportunities but were interested in brief didactic, practice oriented sessions. Both of these groups (non-attenders and less satisfied) indicated more interest than the total study population, in their request for more:

○ on-line learning opportunities,
○ support for small group learning, and
○ personal study (self-assessment, practice audits, personal learning projects

## Discussion

Family physicians reported in the 2010 NPS that they spend on average, 3.09 hours per week or approximately 150 hours per year on CPD activities.^16^ If we include only the 27,000 physicians who are members of the College of Family Physicians of Canada (CFPC) and extrapolate our findings, CPD activities occupy 4,050,000 hours of family physician time yearly. The constant flow of new evidence and recommendations for clinical best practices likely make this time commitment essential, but it is important that physicians can access high quality, reliable resources for their continuing learning that will help them provide optimal patient care.

Overall, our study participants reported being highly satisfied with the learning opportunities available to them and expressed a strong belief that CPD activities do make an important impact on their clinical practices. However, this acceptance of the CPD status quo is challenged by some commentators who believe CPD has to change to create learning activities that result in more substantive improvement in physician performance and patient outcome.^11^,^18^ Many systematic reviews and reports conclude that group learning programs rarely, by themselves, produce changes in practice.^7^,^10^,^19^ Most reviews conclude that it usually takes multiple sources, repetition and a supportive context to move knowledge into practice.^20^ Some commentaries also conclude that physicians may benefit by a more specific “fit” between their personal learning needs and the activities pursued to address these needs.

A recent report recommended that physicians, in order to develop their own individually determined learning plan, need new competencies, such as the ability to assess their own learning needs based on what is happening in their practices; effectively search for and appraise the value of various educational resources; apply those resources to practice-related questions; and use self-assessment and external feedback to evaluate performance within the context of their practice. The professional development process would involve documentation of and recognition for learning and performance outcomes in medical practice.^18^

However, given the low participation reported by our participants in self-assessment, audit or simulation activities, we suspect substantial effort will be required to help physicians appreciate the value and potential impact of these activities. Bodies such as the CFPC and Fédération des médecins omnipraticiens du Québec, which oversee the maintenance of certification of Canadian family physicians, encourage and credit members who develop competencies through small groups and self-directed learning activities. They promote CPD activities such as “linking learning to practice” and self-audits aimed to improve performance and evidence-based patient care.(21) Our survey shows that the group which may be most receptive to these new formats includes people who do not currently attend local CPD events such as workshops and large group didactic sessions.

Offices of CPD in Faculties of Medicine predominantly offer traditional program formats (conferences or courses consisting mostly of large group lecture-style sessions and workshops) which our survey shows, are appreciated by most who responded to our survey. However, the accrediting body for Offices of CPD (a committee of the Association of Faculties of Medicine in Canada) requires that Faculties of Medicine support expansion of opportunities for physicians to explore new options, such as activities that support self-directed learning. Most Offices of CPD are experimenting with new formats in keeping with these trends. In this study, we explored whether physicians are interested or ready for new choices for their continuing education. Although a high proportion of our study participants were regular participants and very satisfied with present offerings, we did detect a group who were not as satisfied and appeared potentially ready to try other formats such as more on-line educational offerings as well as options to better manage the system in which they work.

Our survey has some clear limitations, including the low response rate (31%). The response bias usually introduced by a lower response rate makes it difficult to generalize the results to the full population of general and family physicians practicing in the Champlain Local Health Integrated Network (LHIN). Since it is almost impossible to survey individuals who choose not to participate in surveys, perhaps our expectations regarding response rates for surveys of clinicians needs to adjust to new realities and alternative methods will need to be developed.^22^ Our response rate is higher than many physician surveys e.g. the most recent National Physician Survey, a widely quoted resource reports an 18% rate of return.^16^ We anticipated that it would be difficult to attract “non-participants” in traditional CPD events to do a survey from a traditional CPD provider, and felt fortunate that 39% of our responders were what we defined as “non-attenders”. The use of an incentive to increase participation might have biased the sample towards those who were OCPD “attenders” but the relatively high number of “non-attenders” was reassuring. This enabled us to capture some insights into their preferences. The representative age distributions and their similar responses suggest consistency of our results with the NPS. For example, in terms of learning preferences, our respondents indicated a similar profile regarding group learning and journal reading that continue to be their predominant learning activities. Our participants reported less frequent participation in formal on-line learning programs than the NPS but 45% of our study participants recommended expansion in this area. We conclude that present offerings are not yet meeting the needs of our study physicians but they are optimistic that improvements in on-line learning options could make this choice attractive.

The NPS reported a similar age distribution of physicians practicing in Ontario as ours (67% being over 45 years of age). However, women responders were over-represented (58.4%) compared to our random sample (47%) and higher than the number of women practicing in Ontario (39.3%).^17^ Obtaining CPD needs assessment information from male physicians, who are reported elsewhere as being more likely than women to be non-responders,^22^ remains a challenge for future surveys similar to ours.

Finally, response rates may have been improved if the survey had been conducted electronically. This survey indicated that the majority of sampled family physicians are satisfied with current CPD activities and believe that they are useful. The desire for alternative CPD programs seems greatest in those who are less satisfied with current offerings and indicates a potential audience for new formats such as self-learning, self-assessment and expansion of on-line learning opportunities.

## Figures and Tables

**Table 1:** Characteristics of survey respondents

Demographic and practice characteristics	Response categories	Number of survey respondents*(%)
Gender	Female	87 (58.4%)
	Male	62 (41.6%)
Age category (years)	≤ age 45	48 (32.5%)
	>age 46	100 (67.5%)
Practice type	Solo	8 (5.4%)
	Group	141 (94.6%)
Clinical reimbursement	Salary	18 (12.1%)
	Capitation	52 (34.9%)
	Fee for service	72 (48.3%)
	Not identified	7 (4.7%)
Geographic location of practice	Inner city	22 (14.8%)
	Urban/suburban	81 (54.7%)
	Rural/small town	40 (27%)
	Isolate/remote	1 (0.7%)
	Not identified	4 (2.7%)

* Differences in N values reflect the fact that not all respondents answered each question.

**Table 2:** Current participation in CPD activities, and perceptions of impact of CPD activity on clinical practice (N= 151 responders, out of sample of 485)

CPD activities	Frequent participation (at least “once in a three month period) Number (%)	Most Impact* Number (%)
Live group learning- accredited	*n* = 65 /149 (44%)	*n* = 138/147 (94%)
Live group learning – not accredited	*n* = 65/151 (43%)	*n* = 76/131 (58%)
Rounds, small group activities, journal club	*n* = 90/150 (60%)	*n* = 102/129 (79%)
Peer reviewed journal reading	*n* = 135/151 (70%)	*n* = 113/141 (80%)
Teaching and supervising trainees	*n* = 72/148 (49%)	*n* = 83/119 (70%)
Information seeking using evidence-based resources	*n* = 121/149 (81%)	*n* = 125/145 (86%)

	Infrequent participation (“once/year or never”) Number (%)	Lower impact ** Number (%)

Computer-based education/e-learning	*n* = 75/149 (50%)	*n* = 64/123 (52%)

Self assessment programs	*n* = 105/143 (74%)	*n* = 42/93 (45%)

Practice audits	*n* = 123/144 (92%)	*n* = 28/72 (39%)

Simulation	*n* = 135/144 (94%)	*n* = 17/59 (29%)

* As rated by respondents as “Somewhat significant” or “very significant”* on 5 point scale.

** Lower impact rated as “Somewhat insignificant” or “Very insignificant” impact* on a 5 point scale

**Table 3:** Proposed expanded CPD offerings, presented according respondent age category, conference “attender” category, and level of satisfaction with current CPD offerings

Proposed expanded CPD offerings	Age<46 *n*=45	Age ≥46 *n*=89	*P*	Non-attenders *n*=51	Attended ≥ 1 event *n*=86	*P*	Neutral or less *n*=30	Satisfied or very satisfied *n*=82	*P*
Live accredited large group sessions (>50) expert presentation w/ time for questions	23 (51.1%)	36 (40.5%)	0.285	13 (25.5%)	47 (54.7%)	0.001	11 (36.7%)	41 (50.0%)	0.285
Regional (outside of Ottawa) live accredited course	10 (22.2%)	21 (23.6%)	0.798	14 (27.5%)	19 (22.1%)	0.537	7 (23.3%)	17 (20.7%)	0.798
Brief, didactic presentations (eg.10 mins) practice oriented, within large group sessions	16 (35.6%)	27 (30.3%)	0.520	11 (21.6%)	34 (39.5%)	0.039	13 (43.3%)	30 (36.6%)	0.520
Support for hospital/clinical rounds	11 (24.4%)	17 (19.1%)	0.273	11 (21.6%)	17 (19.8%)	0.829	7 (23.3%)	12 (14.6%)	0.273
Programs that run over several days	8 (17.8%)	11 (12.4%)	0.773	7 (13.7%)	12 (14.0%)	0.970	5 (16.7%)	12 (14.6%)	0.773
A series of sessions such as biweekly, Saturday morning	11 (24.4%)	26 (29.2%)	0.960	11 (21.6%)	26 (30.2%)	0.322	9 (30.0%)	25 (30.5%)	0.960
Support for small group (<15), journal clubs, small group activities, problem based small group learning activities	14 (31.1%)	25 (28.1%)	0.638	18 (35.3%)	21 (24.4%)	0.178	9 (30.0%)	21 (25.6%)	0.638
On-line computer-based, accredited educational programs	19 (42.2%)	41 (46.1%)	0.138	26 (51.0%)	36 (41.9%)	0.375	17 (56.7%)	33 (40.2%)	0.138
Computer based, off-line educational programs (eg. CD-ROM, DVD’s)	6 (13.3%)	10 (11.2%)	0.999	9 (17.7%)	7 (8.1%)	0.106	3 (10.0%)	10 (12.2%)	0.999
Participation in simulations (eg. full/partial task simulators, virtual reality, standardized patients, role play)	10 (22.2%)	24 (27.0%)	0.628	11 (21.6%)	23 (26.7%)	0.545	9 (30.0%)	20 (24.4%)	0.628
Blended learning, some technology, some live elements	12 (26.7%)	19 (21.4%)	0.910	9 (17.7%)	24 (27.9%)	0.217	8 (26.7%)	21 (25.6%)	0.910
Support for personal study (eg. self assessment, practice audit, personal learning projects)	10 (22.2%)	12 (13.5%)	0.361	12 (23.5%)	10 (11.6%)	0.091	6 (20.0%)	10 (12.2%)	0.361
Other	0	1 (1.2%)	0.999	0	1 (1.2%)	0.999	0	1 (1.2%)	0.999
